# (−)-Epicatechin Reduces Blood Pressure and Improves Left Ventricular Function and Compliance in Deoxycorticosterone Acetate-Salt Hypertensive Rats

**DOI:** 10.3390/molecules23071511

**Published:** 2018-06-22

**Authors:** Douglas Jackson, Kylie Connolly, Romeo Batacan, Kimberly Ryan, Rebecca Vella, Andrew Fenning

**Affiliations:** 1 School of Health and Biomedical Science, RMIT University, Bundoora, VIC 3083, Australia; douglas.jackson@rmit.edu.au; 2 School of Health, Medical and Applied Sciences, CQUniversity Australia, Rockhampton, QLD 4071, Australia; k.connolly@cqu.edu.au (K.C.); r.j.batacan@cqu.edu.au (R.B.); k.r.ryan@cqu.edu.au (K.R.); r.vella@cqu.edu.au (R.V.)

**Keywords:** epicatechin, hypertension, DOCA-salt, electrophysiology, cardiac function

## Abstract

(−)-Epicatechin (E) is a flavanol found in green tea and cocoa and has been shown to attenuate tumour necrosis factor alpha (TNF-α)-mediated inflammation, improve nitric oxide levels, promote endothelial nitric oxide synthase (eNOS) activation and inhibit NADPH oxidase. This study investigated the effect of 28 days of low epicatechin dosing (1 mg/kg/day) on the cardiovascular function of deoxycorticosterone acetate (DOCA)-salt hypertensive rats. Wistar rats (n = 120, 8 weeks of age) underwent uninephrectomy and were randomised into four groups (uninephrectomy (UNX), UNX + E, DOCA, DOCA + E). DOCA and DOCA + E rats received 1% NaCl drinking water along with subcutaneous injections of 25 mg deoxycorticosterone-acetate (in 0.4 mL of dimethylformamide) every fourth day. UNX + E and DOCA + E rats received 1 mg/kg/day of epicatechin by oral gavage. Single-cell micro-electrode electrophysiology, Langendorff isolated-heart assessment and isolated aorta and mesenteric organ baths were used to assess cardiovascular parameters. Serum malondialdehyde concentration was used as a marker of oxidative stress. Myocardial stiffness was increased and left ventricular compliance significantly diminished in the DOCA control group, and these changes were attenuated by epicatechin treatment (*p* < 0.05). Additionally, the DOCA + E rats showed significantly reduced blood pressure and malondialdehyde concentrations; however, there was no improvement in left ventricular hypertrophy, electrophysiology or vascular function. This study demonstrates the ability of epicatechin to reduce blood pressure, prevent myocardial stiffening and preserve cardiac compliance in hypertrophied DOCA-salt rat hearts.

## 1. Introduction

Epicatechin, a flavanol found in green tea and cocoa, is being increasingly investigated for its therapeutic potential in metabolic syndrome [1], myocardial ischemia and infarction [2,3,4] and vascular function [5,6,7,8]. In animal models of myocardial infarction and ischemia-reperfusion (I/R), doses as low as 1 mg/kg/day have been beneficial in promoting reductions in infarct size [2,3]. It has also been shown that 10 mg/kg epicatechin intravenously reduced myocardial injury associated with I/R by preserving mitochondrial function in Sprague Dawley rats [9].

Research has shown that epicatechin possesses many cardioprotective and antioxidative properties, including the ability to increase endothelial nitric oxide synthase activation, increasing nitric oxide levels [6,10], and inhibit nicotinamide adenine dinucleotide phosphate (NADPH) oxidase [11], which is a source of cellular reactive oxygen species. It has been suggested that the vascular effects of epicatechin are mediated through opioid receptor activation and importantly that a functional endothelium is required in order for epicatechin to achieve any vascular effects [12]. Additionally, epicatechin has also been reported to attenuate tumour necrosis factor-alpha (TNF-α)-mediated inflammation and insulin resistance [1] and mitochondrial damage [4], all of which have been shown to be associated with myocardial injury. The cardioprotective benefits of epicatechin are not limited to reducing pathological pathways, having been shown to promote phosphorylation of key proteins involved in the physiological remodelling of the heart. These include components involved in the phosphoinositide 3-kinase (PI3K)/protein kinase B (Akt) pathway [13] and both Akt and proto-oncogene tyrosine-protein kinase (Src) [14].

Overall, there is clear evidence of epicatechin’s cardioprotective properties within in vitro experiments and within acute settings such as models of ischemia/reperfusion and infarction, but there has been limited research investigating epicatechin’s ability to preserve cardiovascular functionality within a representative model of chronic cardiovascular disease states such as hypertension and pressure overload. The deoxycorticosterone acetate (DOCA)-salt rat model of hypertension is known to result in significant oxidative stress and cardiovascular remodelling within a relatively short treatment period of four weeks [15,16,17,18,19,20,21,22]. It is therefore a model well suited to investigating compounds that have shown promise in addressing oxidative stress, inflammation and the associated remodelling.

Our study aimed to determine if epicatechin was effective at preserving the functionality of the cardiovascular system within the DOCA-salt rat model of pressure overload hypertension. In doing so, we observed that epicatechin was able to reduce blood pressure, decrease lipid peroxidation and preserve myocardial function despite concurrent hypertrophy of the left ventricle.

## 2. Results

### 2.1. Systolic Blood Pressure

Blood pressure (BP) was significantly elevated in the DOCA group, with an average increase of 64 ± 8 mmHg compared to the uninephrectomy (UNX) controls (Table 1). (−)-Epicatechin (E) treatment significantly reduced BP in both UNX + E (20% lower than UNX) and DOCA + E (24% lower than DOCA) when compared to their respective controls (Table 1).

### 2.2. Biometric Measurements and Serum Malondialdehyde

Rats receiving DOCA-salt injections showed reduced weight gain compared to their UNX counterparts. The mean difference in body mass between DOCA and UNX groups was 93 ± 16 g (Table 1). Cardiac hypertrophy, specifically left ventircular (LV) hypertrophy, was observed in UNX + E, DOCA and DOCA + E groups, with DOCA + E showing the greatest increase in LV mass (Table 1). The left ventricle ratio showed a significant increase in both of the DOCA groups, and two-factor analysis of variance reported that both DOCA and epicatechin treatment had an effect. These results are a surrogate measure for more direct left ventricular hypertrophy analysis; however, they do give an indication that in both the DOCA and DOCA + E rats, there was a significant increase in the size of the left ventricle as a percentage of the total heart weight than the UNX and UNX + E rats. These results confirm the presence of left ventricular hypertrophy in the affected hearts. Both DOCA and DOCA + E had increased kidney and liver mass when normalised to body mass (Table 1).

DOCA-salt control rats showed significantly increased serum malondialdehyde (MDA) concentrations compared to both UNX groups (Table 1). Serum concentrations of MDA had an average increase of 58 pmol/mL within the DOCA group, an increase that epicatechin treatment attenuated (Table 1).

### 2.3. Thoracic Aorta Functional Analysis

**Figure 1:**
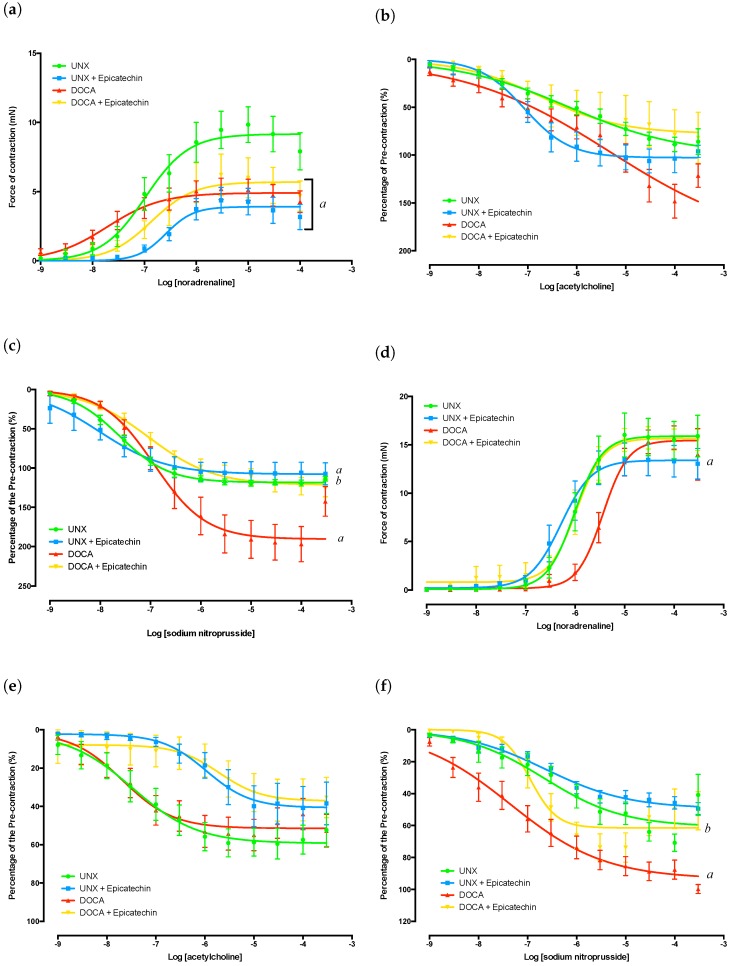
Vascular isolated organ bath experiments. (**a**) Aorta concentration response curve to NA; (**b**) Aorta concentration response curve to ACh following sub-maximal pre-contraction with NA; responses are presented as percentage of pre-contractile force; (**c**) Aorta concentration response curve to NaNO following sub-maximal pre-contraction with NA; responses are presented as the percentage of pre-contractile force; (**d**) Mesenteric concentration response curve to NA; (**e**) Mesenteric concentration response curve to ACh following submaximal precontraction with NA; responses are presented as the percentage of pre-contractile force; (**f**) Mesenteric concentration response curve to NaNO following submaximal precontraction with NA; responses are presented as the percentage of pre-contractile force. ^a^ *p* < 0.05 compared to UNX; ^b^ *p* < 0.05 compared to DOCA.

Aortas isolated from DOCA, DOCA + E and UNX + E groups had significantly diminished responses to noradrenaline (NA)-mediated contraction (Figure 1a) that were accompanied by significant alterations in the effect concentration required to achieve a 50% response (EC_50_ values) (Table 2 and Table 3). Sodium nitroprusside-mediated (NaNO) endothelial-independent relaxation was significantly increased by DOCA-salt treatment, an observation that was attenuated by epicatechin administration (Figure 1c). Epicatechin treatment also impacted on endothelial-independent relaxation in aortas isolated from UNX + E rats, although the effect was not as great as that observed between the DOCA and DOCA + E groups. EC_50_ values for NaNO were significantly affected by both epicatechin and DOCA-salt administration (Table 2 and Table 3).

No significant differences in acetylcholine (ACh)-mediated endothelial-dependent relaxation were observed between any group (Figure 1b). It should be noted that the vasodilation curves have been normalised as a function of the initial pre-contractile force. It can be seen in Figure 1c that the DOCA group relaxed beyond 100% of the pre-contractile force, which is most likely due to the tissue exhibiting a spontaneous increase in vessel tone after pre-tension was achieved yet prior to the zeroing of the channel immediately prior to adrenergic stimuli. Based on this observation, the results of both endothelial-dependent and -independent vasodilation within DOCA groups need to be cautiously interpreted.

### 2.4. Mesenteric Artery Functional Analysis

The contractile function of the mesenteric arteries from DOCA-salt-treated groups (DOCA and DOCA + E) showed no significant difference in their force generation (Figure 1d); however, the DOCA group showed a significant increase in sensitivity to NA as demonstrated by its EC_50s_ (Table 2 and Table 3). This shift in NA EC_50_ values was attenuated in tissue from epicatechin treated animals. The EC_50_ values of NA in mesenteric arteries isolated from DOCA + E rats were comparable to those of the UNX group (Table 2 and Table 3). Epicatechin treatment also caused a significant shift in the NA EC_50_ values in the UNX + E group (Table 2 and Table 3), in addition to an observed reduction in maximal contractile force (Figure 1d).

The EC_50_ values for ACh in epicatechin groups showed a significant decrease in sensitivity of the mesenteric arteries when compared to their untreated counterparts (Table 2 and Table 3). However, there was no difference in the ability of the tissues to relax following NA stimuli. This demonstrates that in both DOCA-salt- and epicatechin-treated groups, there was no significant loss of endothelial-dependent vasodilatory function. It should be noted that none of the ACh concentrations were effective in returning tissues to their resting pre-contracted state. This is likely due to the presence of the initial NA dose still within the myograph chambers. Endothelial-independent function was significantly affected by epicatechin treatment within the DOCA + E mesenteric arteries, but this was not accompanied by a change in NaNO EC_50_ values. It was observed that the DOCA control group demonstrated a much greater vasodilatory capacity than the other tissues (Figure 1f), showing an ability to relax almost back to resting tension following NA stimulation.

### 2.5. Cardiac Function

Electrophysiological impairment was present in the ventricular papillary muscles of both the DOCA and DOCA + E groups, with n action potential durations (APD) showing significant prolongation (Table 4). Measures of APD at 20, 50 and 90% of repolarisation showed a greater than two-fold increase in duration; a change that was not prevented or improved by epicatechin treatment. Neither resting membrane potential, nor action potential amplitude were significantly altered by either DOCA-salt treatment or epicatechin treatment.

Whole heart functional analysis demonstrated the hearts isolated from DOCA control animals had increased diastolic stiffness, as measured by the myocardial stiffness constant (*k*). This change was not observed in the DOCA + E group hearts (Table 4). Rates of contraction and relaxation (+dP/dT and −dP/dT, respectively) and developed pressure were significantly reduced in the DOCA group, indicating a loss of myocardial function and compliance. Treatment with epicatechin was able to attenuate these changes.

## 3. Discussion

The DOCA-salt rat model of hypertension is associated with extensive cardiovascular remodelling, including LV hypertrophy; increased LV interstitial inflammation and collagen deposition; increased diastolic stiffness; and decreased antioxidant and increased superoxide concentrations in the heart and vasculature [16,17,18,21,22,23]. In the current study, epicatechin administration decreased systolic blood pressure in the DOCA + E rats compared to DOCA controls; however, it was unable to fully attenuate the development of hypertension; the blood pressure of the DOCA + E rats was still significantly elevated compared to UNX controls at the end of the treatment period. Notably the reduction in systolic blood pressure was not accompanied by a reduction in heart mass. It can be inferred from this that reduced blood pressure did not have a significant effect on the enlargement of the heart despite improvements in contractile function. This effect on blood pressure is in contrast to a study by Gómez-Guzmán et al. [24] in which treatment of DOCA-salt rats with epicatechin (2 mg/kg/day for 35 days) was not able to significantly reduce blood pressure. The major differences between that experiment and the current study are the age of the rats and the length of the treatment protocol. Gómez-Guzmán et al. [24] started the DOCA and epicatechin protocol when the rats were 14 weeks of age, six weeks older than those used in our study. In addition, their experimental protocol was continued for an additional week, and the impact of additional DOCA stimuli on the pathophysiology of the model cannot be underestimated. Indeed, our study showed epicatechin treatment at 1 mg/kg/day was able to attenuate elevations in serum MDA concentrations by the end of the experimental period, an effect that was only observed at a dose of 10 mg/kg in the study by Gómez-Guzmán et al. [24]. We suggest that the difference in experimental protocol may underpin the observed discrepancy of epicatechin’s effect on serum MDA concentrations.

Although final vasodilatory response to ACh was not significantly different between the groups, there is a clear trend in the UNX + E aortas of showing a much greater reactivity to ACh compared to both the UNX and DOCA + E groups. This is consistent with the findings of Ramirez-Sanchez et al. [6,10] that epicatechin had both endothelial nitric oxide synthase (eNOS)- and NO-enhancing properties. Given that damaged endothelial cells show greater levels of oxidative stress and decreased NO bioavailability, the observation that DOCA + E aortas did not demonstrate a similar trend towards greater ACh sensitivity also indicates the importance of eNOS- and NO-enhancing properties in the vascular effects of epicatechin treatment. Our results are further supported by data from MacRae et al. [12] showing that epicatechin’s vasodilatory effects require a functional endothelial layer, and when the endothelium is damaged or absent, no epicatechin-mediated vasodilation occurs. This helps to explain the difference in epicatechin-mediated effects within the vasculature of both the UNX + E and DOCA + E rats. Vasodilatory response was present and improved within the UNX + E aortas (which possess a functional endothelium), but was not improved in the DOCA + E aortas (which have a significantly damaged endothelium).

The effect of epicatechin treatment on ACh-mediated vasodilation observed within the aortic tissue was not duplicated in the mesenteric tissue, with both UNX + E and DOCA + E showing no significant alteration in final vasodilatory magnitude in relation to pre-contraction. Epicatechin, however, was responsible for a shift in the EC${}_{50}$ of acetylcholine within the mesenteric arteries, an effect most likely mediated via changes in muscarinic receptor expression within the endothelial cells. The M1 and M3 subtypes are the dominant muscarinic receptors expressed within the mesenteric arteries of the rat [25], and therefore, changes in EC${}_{50}$ are likely to be mediated through changes to their expression. However, the underlying mechanisms of this shift in EC${}_{50}$ are beyond the scope of this study, and therefore, we do not discuss them further.

Although epicatechin was unable to prevent hypertension-associated vascular damage, it was able to protect the heart from maladaptive functional changes often associated with enlargement of the myocardium. The presence of hypertrophy within the DOCA-salt rat heart is associated with significant changes to the electrophysiology and whole heart functionality [15,16,18]. Indeed, studies by Fenning et al. [15] and Chan et al. [18] have observed improvements in diastolic stiffness and APD alongside decreased LV hypertrophy in treated DOCA-salt rats. It is therefore of interest that epicatechin was able to improve diastolic stiffness, as well as other measures of whole heart functional compliance, despite no significant improvements in APD or LV mass. As a surrogate measure of LV remodelling, the ventricular volume at a diastolic pressure of 10 mmHg was calculated and found to be significantly increased in both of the epicatechin-treated groups, with the DOCA group showing a reduced ventricular volume at the same pressure. The ratio of LV mass to whole heart mass (LV:WH, where WH is the combined mass of both ventricles) showed a significant increase in both the DOCA and DOCA + E groups. Further, two-factor analysis of variance indicated a trend within the epicatechin groups of higher LV:HW ratios. These results must be interpreted carefully, as they are not direct measures of remodelling or ventricular diameter, but they do support the presence of remodelling and changes to the morphology of the left ventricle. It could be argued that the improved heart functionality is simply a result of reduced haemodynamic stress (driven by a reduction in systolic BP); however, this is unlikely given the sustained LV hypertrophy observed in the DOCA + E hearts. Additionally, the persistence of the APD changes must be considered, as other studies have observed reductions in BP and LV hypertrophy to occur alongside improvements in APD.

The observed disparity between epicatechin’s effect on cardiac function and electrophysiology may be explained by the pathology of the DOCA-salt rat model itself. Electrophysiological changes have been shown to occur before morphological remodelling takes place [26], suggesting that changes in APD may occur early in the pathogenesis of DOCA-salt hypertension before epicatechin’s effects are established. Additionally, prolongation of APD has been attributed to changes in the density of the transient outward potassium current, resulting from cardiomyocyte hypertrophy without a concurrent increase in potassium channel expression [15,27,28]. The presence of cardiac hypertrophy alongside significant reductions in blood pressure provides clear evidence that BP was not the sole, nor the main, driver of hypertrophy within the DOCA-salt hearts. The presence of prolonged APD in the DOCA + E group indicates changes in ion handling were also not affected by either the reduction in blood pressure or epicatechin treatment itself. We are therefore confident that the improvements in diastolic stiffness and other measures of myocardial compliance are a result of changes in the signalling pathways that promote remodelling of the heart. Our study did not measure molecular markers of fibrosis or assess the histology of the groups; however, the ability of epicatechin to alter cardiac remodelling is supported in the literature, with epicatechin showing links to extracellular matrix turnover, particularly via expression of matrix metalloproteinases (MMPs) [2] and cysteine proteases (cathepsins) [29]. Research by De los Santos et al. [13] has also shown the ability of epicatechin to induce physiological hypertrophy.

In their study, De los Santos et al. observed healthy CD-1 mice treated with 1 mg/kg/day epicatechin for 15 days had an 18.7% increase in heart weight to body mass ratio with no evidence of increased fibrosis. They also observed an increase in activation of the PI3K/Akt pathway with Western blot assays, showing increased phosphorylation of associated components [13]. The PI3K/Akt pathway is known to promote physiological hypertrophy within the heart, and the ability of epicatechin to promote such remodelling may explain the presence of hypertrophy within in the UNX + E group. In addition, Panneerselvam et al. [14] has shown that epicatechin can promote phosphorylation of Akt and Src (both important components of cardioprotective pathways) in C57BL/6 male mice [14], further underpinning the potential of epicatechin treatment to promote pro-physiological remodelling.

In conclusion, epicatechin treatment was able to promote a shift in the cardiac remodelling associated with the DOCA-salt rat, and this was a result of the direct action of epicatechin within the tissue and not solely the result of reduced haemodynamic load. This shift in remodelling direction was associated with preservation of cardiac compliance and the prevention of changes of the diastolic stiffness constant; however, epicatechin was not able to prevent changes to the electrophysiology of the DOCA-salt rat heart. We have also provided evidence that epicatechin promoted remodelling within the normotensive rat heart and that this was not associated with detrimental changes in electrophysiological or functional parameters.

## 4. Study Limitations

The current study was designed to assess the functional effects of epicatechin treatment in both a normotensive and hypertensive model, and therefore, it is not designed to determine the mechanism by which the observed functional effects were produced. Further work, including both histological and molecular analysis, is required before any clear mechanistic link can be established between epicatechin treatment and the observed preservation of cardiac function within the DOCA + E animals or the presence of LV hypertrophy within the UNX + E animals.

## 5. Materials and Methods

### 5.1. Chemicals and Treatment

Deoxycorticosterone-acetate (DOCA, D7000, Sigma-Aldrich, Sydney, Australia), *N*,*N*-dimethylformamide (DMF, ≥99%, Sigma-Aldrich, Sydney, Australia), dimethyl sulfoxide (DMSO, ≥99%, Sigma-Aldrich, Sydney, Australia), noradrenaline (99%,), acetylcholine (≥99%, TLC, Sydney, Australia), sodium nitroprusside (≥99%) and (−)-epicatechin (≥90%, HPLC, Sigma) were purchased through Sigma-Aldrich (Sydney, Australia). With the exception of DOCA, which was dissolved in DMF, all other compounds were dissolved in purified water (Milli-Q water purification system, Merk Millipore, Australia). A stock solution of (−)-epicatechin (1 mg/mL) was made up by dissolving 10 mg (−)-epicatechin in a solution of 9.9 mL purified water and 100 μL DMSO.

### 5.2. Establishment of Animal Model

Wistar rats were purchased from the Animal Resource Centre (Perth, Australia) under the approval of the CQUniversity Animal Ethics Committee (Project ID: A11/03-268, Approval Date: 8 March 2011). Upon arrival, the rats were housed within the CQUniversity animal house on a 12-h light/dark cycle and maintained at a temperature of 25 ± 2 ${}^{\circ }$C. They were given free access to water and standard rat chow ad libitum. Once the rats had reached 8 weeks of age and weighed over 300 g, they underwent surgery to remove the left kidney. Uninephrectomy assists with the development of the model by reducing renal function and therefore promoting volume overload. This was achieved by a small flank incision, which exposed the kidney and allowed for the renal vessels and ureter to be ligated. Once ligation was confirmed and blood flow to the kidney had ceased, the kidney was removed by scalpel. The incision was then sutured and the hide stapled. All rats received pain management (Metacam 0.1 mg/kg) on the day of surgery and for two days following surgery. Animals where then randomised into four groups, uninephrectomy only (UNX, normotensive control), uninephrectomy with epicatechin treatment (UNX + E), uninephrectomy with deoxycorticosterone-acetate treatment (DOCA, hypertensive control) and uninephrectomy with deoxycorticosterone-acetate and epicatechin treatment (DOCA + E).

The UNX and UNX + E animals received a subcutaneous injection of 0.4 mL dimethylformamide (DMF, vehicle) every 4th day for the entirety of the experimental period (28 days). This provided a control for any physiological effects associated with the DMF. Animals in the DOCA and DOCA + E groups received subcutaneous injections of 25 mg deoxycorticosterone-acetate salt (dissolved in 0.4 mL DMF) every 4th day for the same 28-day experimental period. The DOCA and DOCA + E groups also had their drinking water replaced with a 1% (*w*/*v*) saline solution for the experimental period.

### 5.3. Experimental Design

In total, 120 rats were distributed across the 4 experimental groups, providing each group with a sample size of 30 animals. The available sample sizes for each experiment were dependent on the requirements of the experiment. The single-cell micro-electrode required the heart to be dissected, preventing it from being used in the Langendorff analysis; therefore, the maximal available sample size for the micro-electrode and Langendorff experiments was 15. Over the course of the experimental period, some animals had adverse reactions and were removed from the study. Epicatechin was administered via oral gavage at a dose of 1 mg/kg/day for 28 days. As an example, a 300-g rat was administered 300 μL of stock solution daily.

### 5.4. Systolic Blood Pressure

Blood pressure was analysed using a modified pressure cuff/pressure transducer system as described by Fenning et al. [15]. Baseline (Week 0) measurements were carried out after the rats recovered from the uninephrectomy and before they were randomised into treatment groups. The baseline results are reported as UNX (Week 0). Representative samples of the groups were assessed at 0, 2 and 4 weeks of treatment. Rats undergoing BP analysis were immobilised by an intraperitoneal injection of tiletamine (15 mg/kg) and zolazepam (15 mg/kg). Once the rats were immobile, a tail pulse transducer (MLT1010) was attached, followed by an inflatable cuff connected to a Capto SP844 physiological pressure transducer (MLT844/D). This was connected to an iMac G4 via a PowerLab 4/30 (ADInstruments, Bella Vista, NSW, Australia). A minimum of 3 blood pressure measurements were taken to produce a mean value from each assessed rat. These data were pooled into treatment groups for statistical analysis.

### 5.5. Serum Malondialdehyde Determination

Blood samples were drawn using a syringe and needle from the thoracic vena cava, following euthanasia, while the heart was still beating and providing adequate circulation. The samples were then transferred to serum separator tubes and allowed to clot before being centrifuged (3000× *g*, 15 min). The supernatant was removed and stored at −80 ${}^{\circ }$C until required for analysis. Serum MDA concentrations were determined using the commercially available OxiSelect^TM^ MDA adduct competitive ELISA kit (Cell Biolabs, Inc, San Diego, CA, USA). Manufacturer’s instructions were followed and a standard curve produced using a 4-parameter logistic curve.

### 5.6. Thoracic Aorta Organ Baths

Following euthanasia and blood sample collection, the thoracic aorta was removed and cleaned while submerged in cold Tyrode’s physiological salt solution (Tyrode’s PSS, mM: NaCl 136.9, KCl 5.4, MgCl${}_{2}$·H${}_{2}$O 1.0, NaH${}_{2}$PO${}_{4}$·2H${}_{2}$O 0.4, NaHCO${}_{3}$ 22.6, CaCl${}_{2}$·2H${}_{2}$O 1.8, glucose 5.5, ascorbic acid 0.3, Na${}_{2}$EDTA 0.05). Five millimetre-long ring segments were then threaded onto stainless steel hooks connected to an FT03 force displacement transducer (Grass Technologies, Middleton, WI, USA). The tissue was anchored in a 30-mL isolated organ bath, filled with warm (37 ${}^{\circ }$C) Tyrode’s PSS and bubbled with carbogen (90% O${}_{2}$/10% CO${}_{2}$). Concentration response curves were performed for NA, ACh and NaNO. Concentration response curves for each tissue sample were normalised as a percentage of their peak (providing a clear 100%), and non-parametric 4-parameter logistical fit analysis was performed to identify the curve and provide an accurate EC${}_{50}$.

### 5.7. Mesenteric Wire Myograph

Mesenteric arteries were assessed using the multi-channel wire myograph system from Danish Myograph Technologies (DMT-Asia Pacific, Bella Vista, NSW, Australia). Second-order mesenteric arteries were dissected and cleaned while submerged in cold Tyrode’s PSS before 2-mm ring segments were mounted in the myograph system. Each sample of mesenteric artery was mounted to the pressure transducer via a 40-μm diameter stainless-steel wire. The tissues were acclimatised for 30 min while the bath temperature was increased to 37 ${}^{\circ }$C, where it was maintained for the entirety of the experiment. Once this temperature was reached, vessels underwent normalisation procedures as prescribed by the manufacturers (DMT, Denmark). After normalisation and a subsequent rest period of 30 min, tissues were subjected to a potassium challenge by rinsing with KCl Tyrode’s (mM: NaCl 37, KCl 100, MgCl${}_{2}$·H${}_{2}$O 1.0, NaH${}_{2}$PO${}_{4}$·2H${}_{2}$O 0.4, NaHCO${}_{3}$ 22.6, CaCl${}_{2}$·2H${}_{2}$O 1.8, glucose 5.5, ascorbic acid 0.3, Na${}_{2}$EDTA 0.05). Once the contractions were achieved, the baths were rinsed with normal Tyrode’s and rested for 30 min. Concentration response curves were performed for NA, ACh and NaNO. EC${}_{50}$ values were obtained using the same method as described previously in the thoracic aorta organ bath methods.

### 5.8. Single-Cell Micro-Electrode

Electrophysiology was performed using the single-cell micro-electrode method described by Fenning et al. [15]. Following the collection of blood, the heart was rapidly dissected and placed in cold Tyrode’s PSS (carbogen gassed). The atria and right ventricle were dissected and the intraventricular septum cut. The least branching papillary muscle was dissected out, pierced at the superior end with a stainless steel hook and the inferior end secured to the base of the electrophysiological experimental chamber (1-mL volume). The chamber was continuously perfused with warm (37 ${}^{\circ }$C), carbogen-gassed Tyrode’s PSS. The papillary muscle was positioned between two platinum electrodes and the hook attached to a modified SensoNor AE 801 micro-force transducer. The transducer was connected to an iMac computer via an amplifier (World Precision Instruments TBM-4). The papillary muscle was then slowly stretched to a pre-load of 5–10 mN over one minute. Electrical field stimulation (Grass SD-9, frequency of 1 Hz, pulse width of 0.5 ms, stimulus strength of 20% above threshold) was used to induce contractions within the tissue. Impalements were made with a potassium chloride-filled (3 M) glass electrode (World Precision Instruments, filamented borosilicate glass, outer diameter 1.5 mm, tip resistance 5–15 mΩ filled with 3 M KCl). Reference readings were provided using a silver/silver chloride electrode. Electrical activity was recorded using a Cyto 721 electrometer (World Precision Instruments) connected to an iMac via a PowerLab 4/25 analogue digital converter. LabChart v5.5 was used to view and record the measurements of the electrophysiology instruments. Each papillary muscle was impaled a minimum of 3 times at three distinct locations, and activity was recorded for a total of 30 min. Recordings were analysed to determine action potential amplitude (APD) at 20, 50 and 90% repolarisation, force of contraction and rate of changes in voltage (dV/dT) and force (dF/dT).

### 5.9. Langendorff Isolated Heart Preparation

Left ventricular function was measured using the Langendorff technique adapted from Chan et al. [17]. Following the collection of blood, the heart was rapidly excised and submerged in ice-cold modified Krebs–Henseleit buffer (modified KHB, mM: NaCl 119.1, KCl 4.75, MgSO${}_{4}$ 1.19, KH${}_{2}$PO${}_{4}$ 1.19, NaHCO${}_{3}$ 25.0, glucose 11.0 and CaCl${}_{2}$ 2.16). The aorta was briefly cleaned of fat, cannulated via the dorsal root and perfused with warm (37 ${}^{\circ }$C), carbogen-gassed modified KHB at a constant pressure of 100 mmHg. A latex balloon catheter connected to a Capto SP844 physiological pressure transducer (MLT 844/D) was inserted into the left ventricle via the mitral valve. The pressure transducer was connected to an iMac G4 computer via a PowerLab 4/30 (ADInstruments, Bella Vista, NSW, Australia), and pressure-volume traces were recorded by LabChart software. The heart was paced at 250 bpm by electrode stimulation of the right atrium, and pressure-volume curves were recorded. Starting from 0 mmHg, the end diastolic pressure was increased in 5 mmHg increments every 60 s until a maximum of 30 mmHg was reached. Diastolic stiffness was assessed by determining the myocardial stiffness constant *k* (dimensionless), obtained by calculating the slope of the linear relationship between stress (σ, dyn/cm${}^{2}$) and tangent elastic modulus (*E*, dyn/cm${}^{2}$) [17]. Other parameters measured were diastolic pressure, developed pressure, left ventricular volume, velocity of contraction (+dP/dT) and relaxation (−dP/dT), as well as end systolic pressure, which were all determined at an intraventricular pressure of 10 mmHg.

### 5.10. Statistical Analysis

Statistical analysis of the data was performed using two-factor analysis of variance and Bonferroni’s post hoc testing. Statistical analysis of diastolic stiffness elevation between the UNX and DOCA groups was performed using a one-tailed Student’s *t*-test. This decision was based on previous literature showing DOCA-salt rats to exhibit increased diastolic stiffness [17], making the directional nature of this test appropriate. Vascular organ bath concentration response curves were analysed by comparing four parameter variable slope nonlinear fits for each group. Comparison of fit analysis was performed with the following hypothesis: null hypothesis was “top same for all datasets” and the alternative hypothesis being “top different for each dataset”. These tests were part of the native statistical analysis offered by GraphPad Prism 6. All statistical analysis was performed using GraphPad Prism 6 software. All data are presented as the mean ± standard error of the mean (SEM). For all tests, *p* < 0.05 was taken as the level of significance.

## 6. Conclusions

We have demonstrated that epicatechin, at a dose of 1 mg/kg/day, is able to promote a shift away from pathological remodelling of the myocardium to a more physiological form of cardiac hypertrophy within the DOCA-salt rat model of hypertension. In doing so, epicatechin treatment preserved whole heart function despite significant hypertrophy and sustained changes in electrophysiological parameters. We have also demonstrated further evidence that epicatechin is able to promote physiological hypertrophy within the hearts of normotensive rats.

## Figures and Tables

**Table 1:** Biometric and haemodynamic measurements.

	UNX	UNX + E	DOCA	DOCA + E
**Body mass (BM) (g)**				
	444 ± 13	452 ± 14	351 ± 10 ^a^	343 ± 15 ^a^
**Organ mass (g/kg BM)**				
Whole heart	2.64 ± 0.14	3.28 ± 0.07 ^a^	3.42 ± 0.12 ^a^	4.01 ± 0.13 ^a,b^
Left ventricle	2.15 ± 0.12	2.74 ± 0.07 ^a^	2.91 ± 0.10 ^a^	3.48 ± 0.12 ^a,b^
Left ventricle ratio	0.81 ± 0.01	0.83 ± 0.01	0.86 ± 0.01 ^a^	0.87 ± 0.01 ^a^
Right ventricle	0.49 ± 0.03	0.55 ± 0.02	0.43 ± 0.05	0.53 ± 0.03
Liver	33.3 ± 0.8	32.8 ± 0.6	37.4 ± 1.4 ^a^	38.6 ± 1.4 ^a^
Kidney	4.88 ±0.10	4.92 ± 0.05	8.31 ± 0.33 ^a^	7.86 ± 0.19 ^a^
Spleen	266 ± 0.11	2.86 ± 0.15	3.11 ± 0.16	3.16 ± 0.38
**Blood pressure (mmHg)**				
0 Weeks	119 ± 6			
2 Weeks	116 ± 6	132 ± 9	162 ± 11 ^a^	134 ± 28
4 Weeks	130 ± 6	104 ± 5 ^a^	194 ± 5 ^a^	147 ± 6 ^a,b^
**Biochemistry**				
MDA (pmol/mL)	125.5 ± 4.1	107.3 ± 8.3	183.6 ± 22.6 ^a^	134.6 ± 4.8 ^b^

Whole heart weight does not include atria, which were dissected off in preparation for functional assessment. MDA, malondialdehyde; E, (−)-epicatechin; DOCA, deoxycorticosterone acetate; UNX; uninephrectomy; ^a^ *p* < 0.05 compared to UNX; ^b^ *p* < 0.05 compared to the DOCA group. Data is presented as the mean value for each group ± the standard error of the mean (SEM).

**Table 2:** Vascular EC_50_ values.

	UNX	UNX + E	DOCA	DOCA + E
**Aorta**				
Noradrenaline	-6.73 ± 0.20	-6.46 ± 0.10	-7.57 ± 0.19	-6.86 ± 0.17
Acetylcholine	-6.35 ± 0.23	-6.83 ± 0.19	-6.26 ± 0.31	-5.81 ± 0.36
Sodium nitroprusside	-7.59 ± 0.11	-8.07 ± 0.14	-6.81 ± 0.12	-6.92 ± 0.15
**Mesenteric**				
Noradrenaline	-5.93 ± 0.14	-6.24 ± 0.12	-5.46 ± 0.10	-6.10 ± 0.25
Acetylcholine	-6.71 ± 0.17	-6.38 ± 0.52	-7.05 ± 0.28	-5.69 ± 0.25
Sodium nitroprusside	-6.27 ± 0.26	-6.66 ± 0.21	-7.20 ± 0.23	-6.28 ± 0.40

EC_50_ values are presented as the mean LogEC_50_ ± SEM. EC_50_ is the concentration required to achieve a 50% response. SEM; standard error of the mean.

**Table 3:** Two-factor ANOVA for vascular EC_50_ values.

	Aorta					Mesenteric				
	DF	SS	MS	F	*p*-Value	DF	SS	MS	F	*p*-Value
**Noradrenaline**										
Epicatechin	1	2.619	2.619	6.917	0.0121	1	2.534	2.534	9.575	0.003
DOCA-salt	1	4.124	4.124	10.89	0.0020	1	1.031	1.031	3.896	0.054
Interaction	1	0.530	0.530	1.400	0.2437	1	0.295	0.295	1.114	0.297
Residual	40	15.15	0.379			44	11.64	0.2646		
**Acetylcholine**										
Epicatechin	1	0.003	0.003	0.003	0.9579	1	9.461	9.461	12.11	0.001
DOCA-salt	1	3.508	3.508	3.925	0.0541	1	0.034	0.034	0.044	0.835
Interaction	1	2.377	2.377	2.660	0.1104	1	1.707	1.707	2.185	0.147
Residual	42	37.53	0.894			41	32.02	0.781		
**Sodium nitroprusside**										
Epicatechin	1	1.052	1.052	5.114	0.0285	1	0.783	0.783	0.897	0.349
DOCA-salt	1	11.23	11.23	54.58	<0.0001	1	0.830	0.830	0.952	0.335
Interaction	1	0.411	0.411	1.998	0.1643	1	4.785	4.785	5.485	0.024
Residual	46	12.39	0.2058			41	35.77	0.872		

DF, degrees of freedom; SS, sum of squares; MS, mean square; *p* < 0.05 assumed significant.

**Table 4:** Electrophysiological and isolated-heart analysis parameters.

	UNX	UNX + E	DOCA	DOCA + E
**Electrophysiological Measurements**				
APD 20% (ms)	11.90 ± 0.56	13.26 ± 1.15	23.77 ± 2.81 ^a^	22.58 ± 4.21 ^a^
APD 50% (ms)	18.63 ± 1.34	22.89 ± 2.79	46.93 ± 6.36 ^a^	45.96 ± 10.40 ^a^
APD 90% (ms)	54.80 ± 5.26	85.11 ± 8.82	120.3 ± 10.51 ^a^	122.1 ± 17.14 ^a^
RMP (mV)	−63.15 ± 4.61	−52.89 ± 4.78	−58.43 ± 3.28	−53.51 ± 4.38
APA (mV)	58.07 ± 3.00	64.48 ± 5.05	60.63 ± 4.78	69.16 ± 5.03
Force (mN)	1.33 ± 0.29	1.33 ± 0.41	1.88 ± 4.88	2.62 ± 0.71
dF/dT (V/s)	0.38 ± 0.08	0.44 ± 0.08	0.52 ± 0.16	0.75 ± 0.18
dV/dT (V/s)	14.19 ± 0.56	16.01 ± 0.48	14.73 ± 1.22	15.30 ± 1.18
**Langendorff Isolated Heart Measurements**				
Diastolic Pressure (mmHg)	10.71 ± 0.45	10.43 ± 0.52	10.78 ± 0.47	9.29 ± 0.34
Ventricular volume (μL)	27 ± 4	48 ± 11 ^a^	22 ± 3 ^a^	44 ± 6 ^a,b^
Developed Pressure (mmHg)	133 ± 17	113 ± 13	81 ± 16 ^a^	131 ± 13 ^b^
+dP/dT (mmHg/s)	2312 ± 336	2067 ± 244	1409 ± 283 ^a^	2580 ± 430 ^b^
−dP/dT (mmHg/s)	−1628 ± 259	−1500 ± 187	−1031 ± 226 ^a^	−1827 ± 167 ^b^
End Systolic Pressure (mmHg)	135 ± 18	123 ± 13	92 ± 16 ^a^	140 ± 12 ^b^
Stiffness (*k*)	30.33 ± 1.28	30.30 ± 2.07	33.93 ± 1.01 ^a^	27.53 ± 1.54 ^b^

APD, action potential duration; RMP, resting membrane potential; APA, action potential amplitude; dF/dT, change in force over change in time; dV/dT, change in voltage over change in time; dP/dT, change in pressure over change in time where the sign indicates the direction of the change; dF/dT values were recorded as the raw output from the force transducer and have not been converted into force units; ^a^ *p* < 0.05 compared to UNX; ^b^ *p* < 0.05 compared to DOCA.

## Abbreviations

ACh	Acetylcholine
DOCA	Deoxycorticosterone acetate
ELISA	Enzyme-link-immunosorbent assay
eNOS	Endothelial nitric oxide synthase
LV	Left ventricular
MDA	Malondialdehyde
MMP	Matrix metalloproteinases
NA	Noradrenaline
NaNO	Sodium nitroprusside
NADPH	Nicotinamide adenine dinucleotide phosphate
NF-κB	Nuclear factor kappa B
NO	Nitric oxide
TLR4	Toll-like receptor 4
TNF-α	Tumour necrosis factor alpha
UNX	Uninephrectomy
